# Collectanea Medica: Consisting of Anecdotes, Facts, Extracts, Illustrations, &c.

**Published:** 1825-05

**Authors:** 


### COLLECTANEA MEDICA:

CONSISTING OF

ANECDOTES, FACTS, EXTRACTS, ILLUSTRATIONS, &c.

*Relating to the History or the Art of Medicine, and the Collateral Sciences.*

Floriferus, ut apes, in saltibus omnia libant,

Omnia nos, itidem, depascimur aurea dicta.

### ART. I.—*Extracts from Elements of Medical Jurisprudence.* By THEODRIC ROMEYN BECK, M.D. &c.

### CHAPTER I.—*Feigned Diseases.*

DISEASES are generally feigned from one of three causes: fear, shame, or the hope of gain. Thus, the individual ordered on service will pretend being afflicted with various maladies, to escape the performance of military duty;—the mendicant, to avoid labour, and to impose on public or private beneficence;—and the criminal, to prevent the infliction of punishment. The spirit of revenge, and the hope of receiving exorbitant damages, have also induced some to magnify slight ailments into serious and alarming illness.

The extent and finish to which the art of feigning diseases is carried, are various, and differ in different countries. Of his own nation, Foderé observes, "that it is at present brought to such perfection, as to render it as difficult to detect a feigned disease, as to cure a real one."

Against such impositions, the police of every well-regulated country should direct its energies. A severe injury may not only be inflicted on individuals through them, but the public morals may be deteriorated. In almost every age, impostors have sprung up, who affect various maladies, and operate on the superstition or the curiosity of the vulgar. And even the higher ranks of society, from motives as unworthy, have occasionally, like the courtiers of Dionysius and Louis XIV., given a sanction to such practices.

It will readily be observed, that a knowledge of this subject may frequently be necessary both in civil and criminal cases, and also in the due administration of Medical Police. To prevent the necessity of repetition, I shall consider it at length under the present division of our subject.

All maladies are not equally capable of being feigned. It is difficult to pretend those whose diagnostic symptoms are certain and established, and whose natural course it is to effect a great change in the system, and to alter the various secretions and excretions in a perceptible manner. But such, on the contrary, as are variable and uncertain in their symptoms, and characterised by little or no change in the external appearance, or where the correctness of an opinion depends much on the statement which the patient may give, are most liable to be feigned. Of the first class may be named inflammations, continued fevers, pu[illegible]lent expectoration, &c.; and of the last, insanity, epilepsy, and pain. Not unfrequently, however, various substances are used to aid in misleading the examiner, and thus the entire skill of a medical man is often called into exercise, to ascertain the real state of the patient.

Zacchias, in his elaborate and learned work, has given five general rules for the detection of feigned diseases, which are so discriminating as to have received the sanction of most succeeding writers. A detail of these will illustrate their universality of application, and the ingenuity of their author.

1. The first is, that the physician must, in all suspected cases, inquire of the relatives and friends of the suspected individual, what are his physical and moral habits. He must ascertain the state of his affairs, and inquire what may possibly be the motive for feigning disease; particularly whether he is not in immediate danger of some punishment, from which this sickness may excuse him. It was on this principle, he observes, that Galen detected the imposture of his servant, who, when ordered to attend his master for a long journey, complained of an inflammation of the knee. He inquired into the habits and character of the slave, and ascertained that he was much attached to a female, whom this journey would compel him to leave. This, combined with the little alteration that so painful an affection as the one named induced, led him to examine the part, and at last to ascertain that the swelling was occasioned by the application of the *thapsia* or *bastard turbith*, and which being prevented, the tumor disappeared.

2. Compare the disease under examination with the causes capable of producing it; such as the age, temperament, and mode of life, of the patient. Thus, artifice might be suspected, if a person in high health, and correct in his diet, should suddenly fall into dropsy or cachexia; and again, if insanity should suddenly supervene, without any of its premonitory symptoms. It is contrary to experience to find such diseases occur without some previous indications.

3. The third rule is derived from the aversion of persons feigning disease to take proper remedies. This, indeed, will occur in real sickness; but it rarely happens when severe pain is present. Any thing that promises relief is generally acceptable in such cases. Those, on the contrary, who feign, delay the use of means. Galen, says Mahon, thus ascertained deceit in another case. An individual complained of a violent colic, on being summoned to attend an assembly of the people. Suspecting artifice, he prescribed only a few fomentations, although this same person had not long before been cured of the same complaint by the use of *philonium*. Of this, however, he never spake, nor indeed seemed in the least anxious for medical aid.

4. Particular attention should be paid to the symptoms present, and whether they necessarily belong to the disease. An expert physician may thus cause a patient to fall into contradiction, and lead him to a statement which is incompatible with the nature of the complaint. To effect this, it is necessary to visit him frequently and unexpectedly.

5. The last direction is to follow the course of the complaint, and attend to the circumstances which successively occur. Thus the inflammation of the knee above noticed should have produced fever, and increased in violence, according to the common course, when no remedies are applied.

Before proceeding to notice separately the various diseases that may be feigned, it will be proper to advert to a species of simulation, mentioned by Zacchias, under the name of ***simulatio latens.*** By this, he understands a case in which disease is actually present, but where the symptoms are falsely aggravated, and greater sickness is pretended than really exists. This may be more difficult of detection in some respects; and it requires, like the cases above noticed, the skill of the physician, and that too of one experienced in the history of disease, to guide aright. Generally speaking, it will be his duty to steer a middle course between too great incredulity and too great confidence; and, where the interests of a third person are not liable to be affected, to lean towards the patient. I can, however, imagine that cases have occurred in which disease has been magnified, in order to increase damages or to revenge insult. Here the conduct of the medical examiner must be cautious, and he should carefully apply the rules already laid down.

The following diseases have at various times been feigned:—Alteration of the pulse; altered state of the urine; hæmaturia; incontinence of urine; suppression of urine; maiming and deformity; dropsy and tumors of various kinds; excretion of calculi and various foreign matters; ulcers; hæmoptysis; hæmatemesis; jaundice and cachexia; fever; pain in various parts; syncope and hysteria; diseases of the heart; apoplexy; paralysis; epilepsy; convulsions; catalepsy; nostalgia; near-sightedness; ophthalmia; blindness and deafness, with or without dumbness.

On examining this list, it will be found that the various diseases are generally referable to two classes,—external infirmities, real in one sense, but arising from factitious and culpable causes; and secondly, internal ones, whose origin is uncertain, and which, from their nature, produce great commiseration on the part of observers.

The *pulse* is sometimes found extremely weak, and occasionally none is perceived at the wrist. Should deceit be suspected, the physician may examine whether ligatures have not been applied to interrupt the pulsation, and he should also ascertain whether the arteries beat at the corresponding extremity. I am indebted to my late worthy preceptor, Dr. M'Clelland, of Albany, for a case illustrating this point. During the period of his attendance at the Royal Infirmary, in Edinburgh, a person applied for and obtained admission on the score of ill health, who had formerly been a patient there. The attending physician examined the pulse at the right wrist, but found none; he then tried the left, but with similar success. The trick was carried on for several days, at the end of which time it was discovered that the patient was in sound health, but that, whenever the pulse was to be examined, he pressed his finger on the artery under the arm-pit.

The *urine* may be altered in many and various ways. The best general rule in suspected cases, is to cause the patient to urinate in the presence of the physician, and to examine the vessel carefully both before and during the process. It is well known that long retention or violent exercise will heighten the colour. Artificial substances have also been used to produce this effect: wine is one; but this may be detected by the smell. Other means are used to give the urine a watery appearance: this symptom, however, should be distrusted, unless other circumstances indicative of disease accompany it. Again, it may be feigned to be bloody. The fruit of the Indian fig *(cactus opuntia)* will colour it as red as blood; and we have a powerful article in the materia medica, viz. cantharides, which will actually produce this state of the excretion, when taken internally. The experiment, however, is too hazardous to be often repeated, even for a temporary purpose. We must also remember that blood may be easily procured, and mixed with it. Hence the importance of the rule already laid down.

*Incontinence of urine.*—Two deserters were brought to the hospital at Martigues, on account of this disease. Foderé was the attending physician, and applied epispastics to the perineum, (a remedy which he in previous cases had found useful,) but without success. They were discharged; but it was shortly discovered that they had feigned the disease. The consequence was an epidemic incontinence of urine among their companions that remained. This awakened the suspicion of our author; and, above all, surprised that his remedy produced no effect in any case, he ordered that the penis of every patient should be tied, and on the knot a seal placed, which none but the gendarme who guarded them should have the power to break, at such times as they wished to urinate. He charged the guard to visit them from time to time, to observe whether the penis was inflated, and also whether the urine was not discharged *guttatim.* He did this from having observed that, in real incontinence of urine, the penis becomes enlarged, so as to render it necessary to remove the ligature in a very short time. The expedient succeeded; it was removed only at the ordinary period, and in twenty-four hours the epidemic vanished.

Dr. Hennen observes, that this disease is almost always detected by giving a full dose of opium at night, without the knowledge of the individual, and introducing the catheter during sleep; or by taking him by surprise during the day, and introducing the same instrument, when it will be found that the urine has not drained off *guttatim* as it was secreted, but that the bladder possesses the power of retention.

*Maiming* is sometimes feigned, under the hope of being admitted into hospitals, such as all high-spirited nations have erected for their wounded and decrepid soldiers and sailors. It may, however, be readily detected by examining the part complained of; as may also *deformity.* In both instances, the part and its articulation should be viewed naked, and compared with the opposite. The following extract from a rare book, called the "History of Knavery," will give an idea of the art that is frequently exercised by beggars. It is a notice published in 1702, and is in the following words:

"That people may not be imposed upon by beggars, who pretend to be lame, dumb, &c. who really are not so, this is to give notice, that the president and governors for the poor of London, pitying the case of one Richard Alegill, a boy of eleven years of age, who pretended himself lame of both his legs, so that he used to go shoving along his breech, they ordered him to be taken into the workhouse, intending to make him a tailor: upon which he confessed that his brother, a boy of seventeen years of age, about four years ago, by the advice of other beggars, contracted his legs and turned them backwards, so that he never used them from that time to this, but followed the trade of begging; that he usually got five shillings a-day, sometimes ten shillings; that he had been over all the counties, especially the west of England, where his brother carried him on a horse, and pretended he was born so. He hath also given an account that he knows of other beggars that pretend to be lame and dumb, and of some that tie their arms in their breeches, and wear a wooden stump in their sleeve. The said president and governors have caused his legs to be set straight; and he has now the use of them, and walks upright."

Of *artificial dropsy and other tumors*, it may be observed, that deception can only escape undetected until the individual be examined by a skilful physician. The following case, from the "Acta Naturæ Curiosorum," will show how much we ought to distrust that affectation of modesty which will not permit a complete investigation:—A young female at Strasbourg, from the enlargement of her abdomen, had led the public to doubt the purity of her character. The distention continued so long as to dissipate the suspicion; and for thirty-nine years she continued to increase in bulk, and excited the commiseration and charity of all who saw her, in such a manner as to lead a highly comfortable life. Her case excited the attention of the physicians and surgeons; and they waited with some impatience until her death should develope the nature of this extraordinary disease. No tumor was found; but in her wardrobe was a sack or cushion weighing nineteen pounds, and so made as to fit the shape of the abdomen. This female would never allow a medical man to examine the seat of her pretended disease.

Sauvages, in his "Nosology," makes mention of a mendicant who gave to his child all the appearances of *hydrocephalus*, by opening the integuments of the head near the vertex, and then introducing air between them and the muscles. This infamous fraud was discovered by removing the patch which covered the hole, and prevented the air from passing out. A mountebank at Brest produced similar inflations, together with the appearance of the most hideous deformity, in a child, by means of the introduction of air, and the application of ligatures on various parts of the body; and not long since a female in France, by the same mode, caused an *emphysema* of the abdominal parietes, so as to resemble dropsy. Tumors of this nature are readily produced, since the cellular texture is spread over the whole surface of the body, and air may be introduced through the smallest possible aperture. We must, however, recollect that dropsy, hydrocephalus, and emphysema, are marked by stronger and more conclusive symptoms than the mere existence of tumor. The sac in *hernia* has been ingeniously imitated with the bladder of an ox; and a *prolapsed rectum* and *uterus*, by means of a portion of the intestine of the same animal, in which a sponge filled with a mixture of blood and milk was placed. It was fixed into the vagina and rectum in such a manner, that one of its extremities was left hanging out.

Chemistry supplies us with the means of ascertaining deceit when the

*excretion of calculi* is feigned. It teaches the characters which designate their animal origin. I should not have noticed this, were it not for a late case related in a highly valuable Journal. A physician was consulted by the friends of a young lady of high respectability, concerning a very painful disease to which she was subjected. She was said to be frequently ill, and during the attack to void, with agonising pain, concretions in her urine. A certain number of these being discharged, she felt relief. A parcel of these urinary concretions was handed to a physician, who instituted experiments on them, and found (what, indeed, was obvious on inspection,) that they were nothing but common sand and pebble stones. Of these, it was asserted she had excreted not less than several pint measures in the course of two or three years. No motives were assigned for this young lady's extraordinary conduct. Dr. Thomson mentions a similar case that occurred in 1811, with a boy about ten years of age.

*Ulcers* are frequently induced by the use of epispastics, or various acrid plants; and real ones are often prevented from healing by similar means. Besides noticing the nature of the discharge, whether it be pus or sanies, and also attending to the habit of the patient, it is sufficient to mention that ulcers caused intentionally are easily distinguished from real ones, since their borders are less callous, their surfaces more superficial, and generally less painful; and by the use of lukewarm water, and covering them with lint, they are readily healed. And the reason for this is, they do not originate from, or accompany, a disease of the system. Frauds of this description are frequently attempted in hospitals, or to avoid the performance of labour of every kind. In 1810, a fellow enlisted in the marines at Portsmouth (Eng.), and received his full bounty. In a few days it was discovered that he had a very bad leg. On investigation, it was proved by his wife and others, that, to avoid going on duty, he had made an incision in the flesh just upon the shinbone, and put a copper halfpenny on the wound, which almost immediately caused a violent inflammation. He ultimately, however, paid most dearly for his speculation, as a mortification followed, and it was found necessary to amputate the limb.

Nor is it confined to common ulcers alone. Even that dreadful disease *cancer* has been feigned. "I have seen," says Pierre Pigray, "a woman present herself to the late king of France, to be touched by him, (as the former kings of France were said to perform miracles in this way,) who appeared to have a very large and ill-looking cancer of the breast. It seemed so extremely natural, that it might have deceived the spectators; but when I observed that she was young, of a good habit, well formed, and without any symptom of cachexia, I was led to suspect deceit. On touching the ulcer, I ascertained, though with some difficulty, that a part of a spleen had been glued on its smooth side, to the nipple, which left on the outside a serous and reddish kind of matter, similar to that of cancer. When this was removed, the nipple remained white, healthy, and well-formed."

A false eruption of *petechiæ* or *pustules* may be detected by examining the patient perfectly naked.

It is easy to feign *hæmoptisis*, by pretending to cough, and then spitting out the blood, which comes from pricking the gums; or it may be assumed by constantly holding some bol. armen. under the tongue, which tinges the saliva of a blood-red colour. Periodical attacks of this disease are most commonly simulated. There are other persons who pretend to be afflicted with *hæmatemesis*, or vomiting of blood, and for this purpose drink the blood of some animal, or use some coloured liquid, and then throw it up in the presence of spectators. Sauvages, in his "Nosology," mentions of a young lady, who, being unwilling to remain in a convent, had some blood of an ox brought to her, which she drank, and then vomited in the presence of her physician. As no deceit was suspected, he stated that she was really ill, and she thus obtained her liberty. A similar case is related of a female, who accused a person of having maltreated her. She went to bed, and brought up large quantities of blood without any effort. She could, however, sing, cry, and put herself in a passion, without the disease recurring, and it ceased when she found that the deceit would prove useless. It will readily occur to the reader that *menstruation* may be pretended, by staining the clothes and body with borrowed blood. All the discharges, however, which I have named, are marked with symptoms which cannot mislead, and need not to be enumerated.

*Jaundice*, when real, is known by the discoloration of the adnata and of the urine. Clay-coloured stools are also another indication; yet it is stated that individuals in France have imitated these to perfection, by taking daily a small quantity of muriatic acid. There are several substances, which, on being taken internally, produce a yellowness of the skin; but in such cases it is proper to recollect that real jaundice is frequently accompanied with vomiting, pain, and sleeplessness. The most unequivocal symptom, and therefore the most to be relied on, is the colour of the adnata. If yellow, jaundice is present, originating either from disease or some artificial cause. *Cachexia* and *great weakness* are also often feigned, by using substances to make the face appear pale and livid. In these instances, inquire whether there is a loss of appetite or of strength, or swelling of the legs. Examine also the pulse and the skin, whether the first be strong and the latter hot.

*Fever* may be induced by the use of various stimulants, such as wine, brandy, cantharides, &c. It is assumed, when a disease is suddenly necessary, to avoid military requisitions, or the performance of work in prisons. Foderé states that he has observed a feverish state of the system thus induced by violent exercise, and then calling for the physician, has noticed the patient imitating the cold fit to admiration. Others, again, having taken emetics to produce similar effects. Of all cases of feigned fever it may be remarked, that they are ephemeral. A day or two's examination developes the deceit, as a frequent repetition of the use of stimulants is too hazardous, and real disease might then be the consequence.

It might appear, from the enumeration already made, that it would require but a moderate share of sagacity to detect feigned diseases. There are, however, some which are very intricate, and they belong to the class previously mentioned, whose origin is uncertain, and where the opinion to be formed depends greatly on the statement to be given by the patient himself.

*Pain* is the disease most frequently assumed; and, in proportion to the facility of assuming it, must be the vigilance of those whose duty it is to detect the fraud. The inquiry should be made, in all suspicious cases, where the disease is seated,—what is probably its cause,—the nature of the pain,—its duration,—its symptoms and effects,—and what remedies have been already used?

The seat of pain is either the external or the internal parts. Patients will not so readily feign the former, since the deceit is liable to be soon detected; and, in addition to this, it is generally of that kind which is deemed a slight disease. Pain in the external parts is, moreover, often accompanied with heat, redness, change of colour, or tumor. Gout is sometimes pretended, and above all rheumatism, for which the soldier is always ready to assign sleeping on the ground as a cause. Both these diseases have diagnostic symptoms,—redness, &c. in the one; and tumefaction or diminution of size, with retraction or loss of motion, in the other. But it is equally true that there are species of severe pain, in which the physician can find no external appearances to found an opinion; and of this description are scorbutic and venereal pains. There are, however, other means of detecting these. Internal pain is accompanied with symptoms which it is impossible to assume, and their absence will, of course, lead to suspicion. Thus, pain in the head is attended with loss of sleep, vertigo, fever, and sometimes with delirium; in the thorax, with cough and difficult respiration; so also in the bowels and kidneys. Each has its peculiar symptoms; which, if the disease be real, are not periodical or occasional in their attack, but incessant, and their severity is generally greater during the night. Inquiry ought also to be made concerning the cause of sickness, and a comparison drawn between it and the violence of the malady. With respect to the species of pain, we should examine whether it be sharp, heavy, or darting, and then compare this with the symptoms. It is, moreover, important to know the duration of the pain complained of; since it is very rare that it is prolonged for any length of time, without exhibiting manifest and unequivocal signs. If violent pain is stated to be present, and the patient, notwithstanding, enjoys a good appetite and sleeps well, we have reason to doubt its severity. Much may also be learnt from the remedies employed. Powerful ones are indicated, if the disease be real, and the patient will not object to their application. It may also be proper to mix a little opium in the food of the patient; and, if sleep be thus readily induced, we may form an opinion as to the magnitude of the disease.

Notwithstanding the above directions, instances have occurred of physicians mistaking real pain for feigned, and feigned for real. "I refused," says Foderé, "for fifteen years, a certificate of exemption to a young soldier, who complained of violent pain, sometimes in one limb, and sometimes in another, and occasionally in the thorax or pericranium, without any external sign to indicate its existence. He died at last in the hospital, from the effects of the malady, which he always insisted was a species of rheumatism. I examined the body after death, viewed all the former seats of disease, but discovered nothing, either in the membranes, muscles, nerves, or viscera; and was hence led to believe that life was destroyed solely from the repetition and duration of these pains." This case induced a determination in our author to be more lenient in future. Its success will be seen in the following instances:—An artillerist, from the garrison of Fort de Bouc, was brought to the hospital at Martigues, with a violent pain in the left leg, and which was attributed to sleeping on the damp ground. During the space of eight months, a variety of antimonial preparations, together with mercurials and tonics, when indicated, were administered, along with local remedies, but without any relief. The leg, from the repeated use of epispastics and cauteries, became thin, and rather shorter than the other; while, from the low diet ordered, there was a general paleness and lankness of the system. Under these circumstances, Foderé could not refuse him a certificate as a real invalid. With the aid of a crutch, he dragged himself to Marseilles, where he obtained the promise of a discharge. He was ordered to return to the fort, to await its arrival; but on his way thither, being too overjoyed, he was met by his commander, walking without his crutch. On being put in prison, he avowed the fraud.

Another case was that of a deserter, a Piedmontese, condemned to hard labour. He was conducted from prison to the workshops, marching on two crutches, as being paralytic in the lower part of the body; and from thence to the hospital, where he remained thirteen months. He supported during that time, with the greatest fortitude, the application of epispastics, moxa, and cupping; asked earnestly for the trial of new remedies, and excited the commiseration of all who saw him. At the end of the above period he was dismissed. In a short time he abandoned the use of his crutches, and never employed them, except when he expected to be observed.

*Feigned syncope* and *hysteria* cannot resist the application of strong sternutatories to the nostrils. In the former, it is difficult to dissemble a small, feeble, and languishing pulse, an almost suppressed respiration, cold sweats, coldness of the extremities, and great paleness of the countenance. If ligatures are supposed to be used to prevent the pulse being felt, the body should be examined naked. So also, if lotions have been applied to the face to give it a pale colour, let the face be washed. The causes assigned for producing the disease, and the rapidity with which the symptoms have presented themselves, should also be noticed.

Cases are, however, mentioned, where individuals possessed the power of suspending, or at least moderating, the action of the heart: and, on the contrary, some have been able to increase it at will. All such require patient and repeated examinations.

*Apoplexy* will only be feigned by those who hope for immediate escape from some impending punishment. From the nature of the disease, it cannot be long dissembled. If it be necessary to ascertain the truth at the first moment of the attack, powerful remedies (such indeed as are indicated in the real disease) should be employed. Zacchias observes, that feigned apoplectics cannot resist the action of sternutatories. *Paralysis*, in many respects, requires the same treatment as rheumatism. A powerful shock from an electric jar may tend to develope the deceit.

It is remarkable that a disease so much dreaded by the real sufferer as *epilepsy*, should be so often feigned; yet this is really the case: and the cause probably is, the affright and pity that may be inspired, or else the short exhibition of disease that is necessary, leaving the patient to act as he pleases during the interval. In all suspicious cases, it is proper to notice whether the sick person falls to the ground suddenly; whether the face is livid, the pupil fixed, the lips pale, the mouth distorted and frothy, and the pulse altered. The physician ought also to observe whether sleep follows the paroxysm; and also if the patient complains of dulness of sensation, vertigo, and great weakness. All or most of these symptoms accompany real epilepsy. But the surest sign of this disease is a loss of feeling, so that sternutatories, and even the actual cautery, produce no effect during the paroxysm: this immediately gives us a mode of detecting artifice. An artillerist at Martigues had acquired, from frequent practice, such skill in feigning this disease, as almost to deceive Foderé; and this, indeed, would have been the case, had he been able also to resist the application of fire. This always recovered him, though he lay apparently without sense, his eyes starting from their orbits, and his mouth foaming. He afterwards confessed that he never counterfeited a paroxysm, without feeling for several days a violent pain in the head.

De Haen was consulted by a mother, whose daughter, after being cured of deafness, became epileptic. He directed her to be brought to the hospital at Vienna, where he attended. The fit, which at first did not occur more than once or twice a-day, now recurred every hour. It resembled a real one, as the hands were violently clenched, and the eyes disordered; but he suspected deception, for the following reasons:—she did not open her eyes during the paroxysm with a wink, but in the natural manner; her pulse was natural; when the curtains were drawn, the pupil of the eye was dilated, and when opened it was contracted, and this last occurred very violently when a candle was presented. Convinced that the disorder was pretended, he ordered her to be taken out of bed, and directed the attendants to keep her in an erect posture. If she fell, they were to chastise her severely. A cure was thus effected, and she confessed that both the deafness and epilepsy were feigned, to avoid going to service. In another case, a female, aged twenty, was confined in prison for a murder, who had on her the marks of three successive burnings, which she resisted without confessing the deceit. De Haen, and many others, saw her imitate a paroxysm of epilepsy with such horrible accuracy, that the feigned was supposed to be real, until, in the midst of it, being ordered to rise, she got up and walked away. In such an instance, our author recommends the remedy used at Paris. A beggar there often fell into fits in the street: a bed of straw, through compassion, was prepared, on which he might be laid to prevent injury to himself; when next attacked, he was laid on it, and the four corners set on fire; he sprang up, and fled.

One fact should be kept in mind respecting this disease. The real epileptic is desirous of concealing his situation, and attaches to it a kind of false shame; while the feigned talks about the disease, and takes no precaution to avoid publicity.

*Convulsions,* when feigned, do not present that stiffness of the muscles, or that resistance and rapidity of action, which appear in the real. The treatment must be similar to that of epilepsy. "Twenty years ago," says Foderé, "I proved, by the aid of fire and force applied to the antagonist muscles, that a woman, who had imposed on a good curate in the Alps, was an impostor. She was supposed to be possessed, fell down apparently without sense, and made frightful contortions. She could not, however, withstand the above tests, and rose up, to her great confusion and the astonishment of the spectators."

Similar proofs ought to be applied to those cases of *chorea* which are not unfrequently seen in our streets. It has been satisfactorily ascertained that, in some instances, the disease is assumed to impose on public beneficence.

*Ecstacies* and *possessions* are now with justice considered impossible, and those who pretend to them as impostors. There is, however, one form of disease, nearly allied to those we are noticing, which deserves some attention. I allude to *catalepsy.* Its nature is but little understood; but we are informed that the patient is suddenly motionless, while the joints remain flexible, and that external objects make no impression. There seems no reason to doubt that real cases have existed; but, if there be any cause for suspicion, the remedies already indicated should be administered. The state of insensibility which some have feigned, might possibly, if real, be referred to this. Dr. Smith makes mention of a soldier, named Drake, who assumed an appearance of total insensibility, and resisted for months every sort of treatment,—even the shower-bath and electricity; but, on a proposal being uttered in his hearing to apply red-hot iron, his pulse rose, and an amendment shortly took place. The case of Phineas Adams, which lately occurred in England, shows to what individuals will submit, in order to escape punishment. He was a soldier in the Somerset militia, aged eighteen years, and confined in gaol for desertion. From the 26th of April to the 8th of July, 1811, he lay in a state of insensibility, resisting every remedy,—such as thrusting snuff up the nostrils, electric shocks, powerful medicines, &c. When any of his limbs were raised, they fell with the leaden weight of total inanimation. His eyes were closed, and his countenance extremely pale; but his respiration continued free, and his pulse was of a healthy tone. The sustenance he received was eggs diluted with wine, and occasionally tea, which he sucked in through his teeth, as all attempts to open his mouth were fruitless. Pins were thrust under his finger-nails to excite sensation, but in vain. It was conjectured that the present illness might be owing to a fall; and a proposal was consequently made by the surgeon to perform the operation of scalping, in order to ascertain whether there was not a depression of the brain. The operation was described by him to the parents, at the bed-side of their son, and it was performed: the incisions were made, the scalp drawn up, and the head examined. During all this time he manifested no audible sign of pain or sensibility, except when the instrument with which the head was scraped was applied. He then, but only once, uttered a groan. As no beneficial result appeared, and as the case seemed hopeless, a discharge was obtained, and he was taken to the house of his father. The next day he was seen sitting at the door, talking to his parent; and the day after was observed, at two miles from home, cutting spars, carrying reeds up a ladder, and assisting his father in thatching a rick.

*Nostalgia*, or *maladie du pays*, is a disease common in military hospitals. This mental affection, if carried to excess, soon produces a physical one, and a mixed state is produced, in which all the marks of melancholy and hypochondriasis are visible. Young men are more subject to it than persons advanced in life,—villagers more than citizens; and, among nations, it is found to prevail most in the Swiss, the Savoyards, the inhabitants of the Pyrenees, the Flemings, &c. Besides the above considerations, and that alteration of countenance which it is impossible to feign, it may be added, that "pretenders generally express a great desire to revisit their native country; whilst those who are really diseased are taciturn, express themselves obscurely on the subject of their malady, dare not make an avowal, and are little affected by the consolations which hope or promises offer to them."

"It is curious to observe," says Foderé, "how many young men have, during the last twenty years, worn convex glasses, in order to acquire *myopia*, or *near-sightedness;* which, however, is not the certain consequence, but more commonly this practice leaves a weakened and defective sight, differing from it, and also from that which is the effect of old age. It is not from an inspection of the eye, nor from the account of the individual, that we can judge concerning the reality of the complaint; but it may be ascertained by presenting an opened book, and applying the leaf close to the nose, or by putting on glasses proper for near-sighted persons. If the individual cannot read the book distinctly when placed thus, or when the above glasses are used, we may feel confident that his disease is feigned." This mode of examination should be strictly adhered to; since, as far as my observation has extended, no complaint is more frequently urged by those who wish to avoid military duty, than near-sightedness.

*Ophthalmia* has often been artificially excited, by the application of various stimulant remedies. It is, however, readily detected by the rapidity of its progress: it arrives at its acme within a few hours after the application of the acrid substance. Some information may also be derived from noticing which eye is affected. A few years since, when an extensive system of deception prevailed in the British 28th regiment of Foot, Dr. Vetch observed that the counterfeit inflammation was almost solely confined to the right eye. A left-handed man would probably inflict the injury on the left eye.

